# CB1 Receptor Activation Provides Neuroprotection in an Animal Model of Glutamate‐Induced Excitotoxicity Through a Reduction of NOX‐2 Activity and Oxidative Stress

**DOI:** 10.1111/cns.70099

**Published:** 2024-11-04

**Authors:** Ari Misael Martínez‐Torres, Julio Morán

**Affiliations:** ^1^ División de Neurociencias, Instituto de Fisiología Celular Universidad Nacional Autónoma de México Ciudad de México Mexico

**Keywords:** AQP4, CB1, excitotoxicity, glutamate, NOX‐2, reactive oxygen species

## Abstract

**Background:**

Excitotoxicity is a process in which NADPH oxidase‐2 (NOX‐2) plays a pivotal role in the generation of reactive oxygen species (ROS). Oxidative stress influences the expression of Aquaporin 4 (AQP4), a water channel implicated in blood–brain barrier (BBB) permeability and edema formation. The endocannabinoid system is widely distributed in the brain, particularly through the cannabinoid receptor type 1 (CB1) and type 2 (CB2), which have been shown to have a neuroprotective function in brain injury. Given the significant involvement of NOX‐2 in ROS production during excitotoxicity, our research aims to assess the participation of NOX‐2 in the neuroprotective effect of the cannabinoid receptor agonist WIN55,212‐2 against glutamate‐induced excitotoxicity damage in the striatum using in vivo model.

**Methods:**

Wild‐type mice (C57BL/6) and NOX‐2 KO (gp91^Cybbtm1Din/J^) were stereotactically injected in the striatum with monosodium glutamate or vehicle. Subsequently, a group of mice was administered an intraperitoneal dose of WIN55,212‐2, AM251, or AM251/WIN55,212‐2 following the intracerebral injection. Motor activity was assessed, and the lesion was examined through histological sections stained with cresyl violet. Additionally, brain water content and Evans blue assay were conducted. The activity of NOX was quantified, and the protein expression of CB1, gp91^phox^, AQP4, Iba‐1, TNF‐α, and NF‐κB was analyzed using Western blot. Furthermore, ROS formation was measured through the DHE assay.

**Results:**

The activation of the endocannabinoid receptors demonstrated a neuroprotective response during excitotoxicity, meditated by NOX‐2. The reduction in ROS production led to a decrease in neuroinflammation, and AQP4 expression, resulting in reduced edema formation, and BBB permeability.

**Conclusions:**

During excitotoxic damage, WIN55,212‐2 inhibits NOX‐2‐induced ROS production, reducing brain injury.

## Graphical abstract

During excitotoxicity, reactive oxygen species, produced by NADPH‐oxidase 2 (NOX‐2), increases the levels of aquaporin‐4 (AQP4) leading to the rupture of the blood–brain barrier and cerebral edema. This along with oxidative stress contributes to neuronal death. Stimulation of cannabinoid receptor type 1 receptors relieves oxidative stress by a reduction of NOX‐2 activity and thus inhibits AQP4 increase, edema, and brain damage. The endocannabinoid system can be a therapeutic target for limiting brain damage.

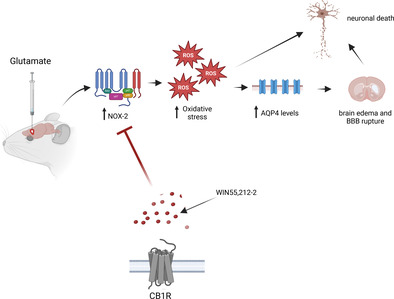

## 1 Introduction

The imbalance between glutamate release and reuptake results in a neurotoxic condition in neurons known as excitotoxicity, usually present in both acute and chronic brain disorders [1]. The overstimulation of glutamate receptors causes elevated levels of intracellular calcium and reactive oxygen species (ROS), along with the activation of microglia and subsequent inflammation [2]. The increase in ROS production is a significant factor contributing to the advancement of damage and eventual neuronal death [3].

One of the ROS sources is the NADPH oxidases family (NOX), particularly NOX‐2, which is widely expressed in neurons, astrocytes, and microglia, and is implicated in neurological disorders [4, 5]. Our research has shown that during excitotoxicity, the absence or inhibition of NOX‐2 leads to a reduction in neuronal death, ROS production, lesion size, and improvement in motor recovery [6, 7]. After the brain injury, the pathological accumulation of fluid, known as edema, contributes to neuronal death [8]. Notably, excitotoxicity plays a pivotal role in the initial stages of edema development, especially before the disruption of the blood–brain barrier (BBB), a critical event associated with neuronal death [9]. Aquaporin 4, the primary water channel in the brain, is essential for BBB function and is predominantly located in the endfeet of astrocytes, specialized glial cells that interact with brain blood vessels [10]. Increased levels of AQP4 are associated with increased edema and BBB disruption after brain injury, while the inhibition or elimination of AQP4 activity seems to provide neuroprotection [11, 12]. Various molecular mechanisms, including oxidative stress, are involved in the regulation of AQP4 expression [13, 14].

The Endocannabinoid System (ECS) comprises the cannabinoid receptor type 1 (CB1) and type 2 (CB2), the endocannabinoids arachidonoyl ethanol amide (anandamide) and 2‐arachidonoyl glycerol (2‐AG), and the enzymes responsible for their synthesis and degradation [15]. While ECS is known for its involvement in neurodevelopment and plasticity, it has also been associated with the protection of the brain against acquired injuries and neurodegenerative pathologies [16]. Cannabinoid receptors are 7‐domain membrane G protein‐coupled receptors, with CB1 being the predominant receptor in the brain [17].

As mentioned earlier, ECS exerts a neuroprotective function in cases of brain injury. In an ischemic stroke model, the stimulation of CB1 receptors using arachidonyl‐2′‐chloroethylamide (ACEA), a CB1 agonist, resulted in a decrease in the infarct area and enhancement in motor recovery. This effect was reverted with the co‐administration of AM‐251, a CB1 antagonist [18, 19]. Furthermore, studies have demonstrated that the administration of 2‐arachidonoylglycerol (2‐AG) reduced the injury area and neurological impairment while preserving the integrity of the BBB after traumatic brain injury (TBI) [20, 21]. Another synthetic agonist, WIN55‐212‐2 (WIN55), administered orally or intraperitoneally in rats subjected to permanent middle cerebral artery occlusion (MCAO), reduced brain edema and infarct volume [22, 23].

The role of ECS in the regulation of oxidative stress in neuronal injury has been recently evaluated. The CB1 stimulation with WIN55, in a model of quinolinic acid‐induced neurotoxicity in primary rat striatal cell cultures, prevented lipid peroxidation, ROS formation, and decreased cell viability [24]. Also, the inhibition of anandamide degradation by URB597 increased catalase and SOD activity and reversed the increases of ROS and malondialdehyde (MDA) in BMEC cell cultures exposed to oxygen–glucose deprivation [25]. In an animal model of chronic unpredictable stress, the increment in anandamide levels also reduced lipid peroxidation [26]. Comparable results were reported using ACEA in zebrafish subjected to acute restrain stress (ARS) [27, 28].

Several studies provide evidence that the stimulation of the ECS produces an antioxidant and neuroprotective effect; however, the precise involvement of NOX‐2 and AQP4 in ECS‐induced neuroprotection remains unclear. This study aimed to evaluate the role of oxidative stress, NOX‐2, and AQP4 in the neuroprotective effects of WIN55, a CB1 and CB2 mixed agonist in an in vivo excitotoxicity model. This was done by examining motor activity, lesion area, NOX activity, brain edema, BBB integrity, AQP4 levels, and ROS production.

## 2 Materials and Methods

### 2.1 Animals

Animals were managed according to the National Institute of Health Guide for the Care and Use of Laboratory Animals (NIH Publication No. 8023 revised 1978) and the Local Committee for the Care and Use of Laboratory Animals (CICUAL protocol JMA 182‐22). Efforts were made to minimize pain, and the number of animals used. Wild‐type (WT) adult mice C57BL/6 (8 weeks old) were obtained from the bioterium of the Instituto de Fisiologia Celular, Universidad Nacional Autonoma de Mexico. NOX2 KO adult mice were generated on a C57BL/6 background and were purchased from the Jackson Laboratory (Bar Harbor, ME), the colony was established in the vivarium of the Instituto de Fisiologia Celular, Universidad Nacional Autonoma de México. Mice were housed and kept under controlled temperature (20°C–22°C) with a regulated 12‐h light–dark cycle, with water and food as libitum.

### 2.2 Intrastriatal Glutamate Injection

Animals were anesthetized with ketamine/xylazine 80/5 mg/kg intraperitoneally (i.p.) and placed on a stereotaxic frame. A stainless‐steel needle was positioned in the right striatum according to the coordinates anteroposterior +0.8 mm from bregma, lateral +2.2 mm from midline, and vertical 3.2 mm from the dura. Animals were treated as indicated in Table 1. Using an injection pump (KDS 210 Kd Scientific), 0.5 μL of a solution of monosodium glutamate (1 M) (GLUT) or vehicle (saline solution 0.9%) (CONTROL) was injected (0.175 μL/min). At the 5‐min mark postinjection, the needle was removed with care, and the incision was sealed using DERMABOND ProPen. After the procedure, mice were given an intraperitoneal injection of either AM251 (GLUT+AM251) or WIN55 (GLUT+WIN55) at 2 mg/kg [29, 30, 31]. The drugs were administered immediately after glutamate intracerebral injection to enhance their effectiveness during excitotoxic lesions. AM251 was selected for its selectivity for CB1 receptors in the striatum and its brain availability [32, 33]. A subset of animals was pretreated with AM251 30 min prior to the intracerebral glutamate injection, while WIN55 was administered immediately after glutamate treatment (GLUT+AM251/WIN55). Drugs were dissolved in dimethyl sulfoxide (DMSO) and diluted in saline. The control groups were treated with the same vehicle. Mice recovered from anesthesia in a temperature‐controlled chamber and were placed in individual cages with water and food ad libitum.

**TABLE 1:** Experimental groups in WT and NOX‐2 KO animals.

Groups	Intrastriatal injection	Treatment (i.p.)
CONTROL	Saline solution 0.9%	Saline solution 0.9%
GLUT	Glutamate 1 M	Saline solution 0.9%
GLUT+WIN55	Glutamate 1 M	WIN55,212‐2 immediately after intrastriatal injection 2 mg/kg
GLUT+AM251	Glutamate 1 M	AM251 immediately after intrastriatal injection 2 mg/kg
GLUT+AM251/WIN55	Glutamate 1 M	AM251 30 min before injection and WIN55,212‐2 immediately after injection

A subset of animals (*n* = 7) was chosen at random to undergo motor activity tests at 1, 6, 12, and 24 h post‐surgery. Subsequently, these same animals were randomly assigned to groups for analysis of lesion volume, cerebral water content, and BBB permeability assay. This experimental setup was intended to reduce the total number of mice required for the study. The rest of the animals were allocated to the remaining experiments, with each animal participating in only one distinct experiment (*n* = 3).

The selected time points for the analysis of lesion volume, cerebral water content, and BBB permeability were based on previous studies demonstrating that excitotoxic injury in the striatum becomes apparent at 12 and 24 h following the intracerebral injection of glutamate [6, 7].

### 2.3 Cylinder Test of Forelimb Asymmetry

Animals were examined for preferential use of the unilateral forelimb during upright postural support before and after glutamate intracerebral injection [34]. Mice were placed in a glass cylinder (11 cm in diameter) on a transparent tabletop and evaluated for 3 min. The number of unilateral and bilateral wall contacts was recorded. The percentage of bilateral contacts was assessed on each test using the formula 100× bilateral contacts/total forelimb wall contacts, and the percentage of unilateral contacts was assessed using the formula 100× unilateral contacts/total forelimb wall contacts. The results are expressed as the percentage of unilateral exploration.

### 2.4 Adhesive Removal Test

Animals were introduced to the test environment half an hour early to acclimate. They were carefully taken from their cages, and a small, half‐centimeter square of adhesive tape was affixed to the top part of their snouts before being placed back into their cages. Observations were made and notated for 60 s or until the animals removed the tape with their front limbs. The animals underwent training for five consecutive days, one session daily, and were assessed post‐glutamate injection. The study quantified the duration required for the animals to remove the tape, documenting this interval in seconds [35].

### 2.5 Inverted Grid Test

This test was designed to evaluate limb strength and coordination [36]. Mice were situated in the middle of a wire mesh grid, which measured 20 × 20 cm, with a mesh size of 0.5 cm^2^. The grid was bordered by walls made of wood. The grid was placed 20 cm above a tabletop and was rotated. Mice were observed for 60 s. No pretraining was performed, but a pretest of 30 s before the day of the experiment was conducted for acclimatization.

### 2.6 NOX Activity

NOX activity was determined in striatal homogenates in lysis buffer at 1, 6, 12, and 24 h after glutamate intracerebral administration and it was estimated as the oxidation of dihydroethidium (DHE) to ethidium (Et). Tissue homogenates were incubated with 0.02 mM DHE (Sigma, 37,291), 0.5 mg/mL salmon DNA (Behringer, 1,146,714), and 0.2 mM NADPH (Sigma, N7505) as substrate. Et fluorescence was measured during 30 min at 480 nm and emission of 610 nm using a Synergy HT Multi‐Detection fluorescence microplate reader (Biotek Instruments, Colchester, VT). Samples were prepared in duplicates, and NOX activity was quantified by the alteration in Et fluorescence per milligram of protein per minute in comparison to the control.

### 2.7 Western Blot

At 1, 6, 12, and 24 h after surgery, animals were anesthetized, and killed by decapitation and the striatum was dissected and homogenized. The tissue obtained (10–20 mg) was homogenized in RIPA buffer and protease inhibitors at 4°C (OMNI TC homogenizer). Homogenates were centrifuged at 5000 × *g* for 5 min, and protein concentrations were determined using a DC Protein Assay kit (BIO‐RAD, cat# 5000111) according to the manufacturer's instructions. Protein homogenates of 50 μg per lane were loaded onto a 12.5% native gradient gel subjected to SDS‐PAGE and then transferred onto a PVDF membrane in Tris‐glycine‐methanol transfer buffer at 100 V for 75 min at 4°C. The membranes were blocked overnight with 6% nonfat dry milk in PBS and incubated for 2 h with anti‐gp91phox (1:5000; 12,906, Abcam, USA), anti‐AQP4 (1:3000, sc‐32,739; SCBT, USA), anti‐Iba1 (1:10000, sc‐32,725; SCBT, USA), anti‐CB1 (1:5000, sc‐518,035; SCBT, USA), anti TNF‐α (1/5000, sc‐52,746, SCBT, USA) anti NF‐ΚB p65 (1/2000, ab32536, Abcam, USA), and anti‐GAPDH (1/5000, 14C10, Cell signaling, USA) antibodies. After washing, membranes were incubated with alkaline phosphatase‐conjugated secondary antibody (1:50000) for 1 h. The protein bands were visualized using a C‐DiGit Blot Scanner (LI‐COR, USA). Initially, the blots were probed for GAPDH antibody. Following this, they were stripped and subjected to probing with additional primary antibodies. The outcomes were presented as the proportion of the protein of interest to GAPDH.

### 2.8 Brain Water Content

At 12 and 24 h after surgery, animals were anesthetized and decapitated. Brains were extracted and weighed. Subsequently, the tissue was dried at 100°C for 24 h, and the dry weight was documented. The percent of water content on each hemisphere was calculated using the following equation [37]:

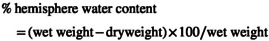




The individual percentage of change among hemispheres was calculated utilizing the equation:

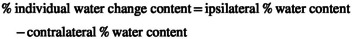




### 2.9 Brain–Blood Barrier (BBB) Permeability

After 24 h of the intracerebral injection, mice were administered with 2% Evans blue solution (Sigma, E2129) at a dose of 4 mL/kg, i.p. After 1 h, the mice were infused with 80 mL of 1X PBS. The brains were extracted and the tissue corresponding to the right striatum was dissected. Tissue was weighed and homogenized in 1 mL of 1X PBS, vortexed, and 1 mL of trichloroacetic acid (Sigma, T915) at 60% was added. Samples were kept at 4°C for 30 min, centrifuged at 16,000 rpm, and 250 μL of the supernatant was placed on a microplate. Evans blue concentration was measured at a wavelength of 610 nm, and content was calculated from a standard curve derived from the dye, and results were expressed in μg/mg of tissue [38].

### 2.10 Lesion Volume Analysis

Glutamate‐induced lesions were evaluated at 24 h after intracerebral injection. Mice were anesthetized with ketamine/xylazine and were transcardiacally perfused with 40 mL of a 0.9% saline solution followed by 40 mL of 4% paraformaldehyde solution in 0.1 mM phosphate buffer at pH 7.4. Brains were removed and placed in the same fixative solution at 4°C. Consecutive series of coronal sections (40 μm thick) for cresyl violet were obtained in a cryostat (1510s, Leica, Microsystems Nussloch GmbH, Heidelberg, Germany). Upon completion of the staining process, any sections displaying a lesion were chosen to measure the volume of the lesion. The areas of damage were outlined by hand and quantified with the help of an image analysis program (Image J version 1.48v; Wayne Rasband, National Institutes of Health, USA) by a researcher who was unaware of the treatment details. The overall volume of the lesion was calculated by adding the volumes of all the damaged sections, each multiplied by the thickness of the slices, which was 40 μM.

### 2.11 ROS Determination

ROS levels were evaluated in WT animals at 1, 3, and 24 h after glutamate intracerebral injection. Mice were intraperitoneally administered with dihydroethidium solution (DHE) (Sigma‐Aldrich, 37,291) at a dose of 25 mg/kg solubilized in 100% DMSO 30 min before intracerebral injection and sacrifice. Mice were transcardiacally perfused as described above. Brains were stored at 4°C and transferred successively to 20% and 30% sucrose solution for cryoprotection. Coronal 25 μm sections were obtained in a cryostat, mounted, and stained with Hoechst stain (Invitrogen, 33,342) for 15 min. Slides were washed and covered with Fluoromont‐G (Invitrogen, 00‐4958‐02) and maintained at 4°C until examination. Preparations were observed under an epifluorescence Olympus microscope using a U‐MNG2 filter (universal narrow green, 528–605) and a U‐MNU filter (universal mirror narrow ultraviolet, 355–465) for Et and Hoechst, respectively. Et fluorescence was quantified (Image‐Pro Plus analyzer program) in the entire striatum in three sections per mouse. Data is expressed as arbitrary fluorescence units.

### 2.12 Statistical Analysis

Data are presented as the mean ± standard deviation. Statistical analysis was conducted using GraphPad Prism version 8.0 (California, USA). The Shapiro–Wilks test was used to assess data distribution. The data with a normal distribution underwent analysis of variance with further post hoc analysis using Tukey's test for multiple comparisons. The data without normal distribution was analyzed with a nonparametric equivalent Kruskal–Wallis with further post hoc analysis using Dunn's test for multiple comparisons. A *p* < 0.05 was considered to indicate statistically significant differences.

## 3 Results

### 3.1 Glutamate Injection Increases CB1 Protein Levels in WT Mice

**FIGURE 1:**
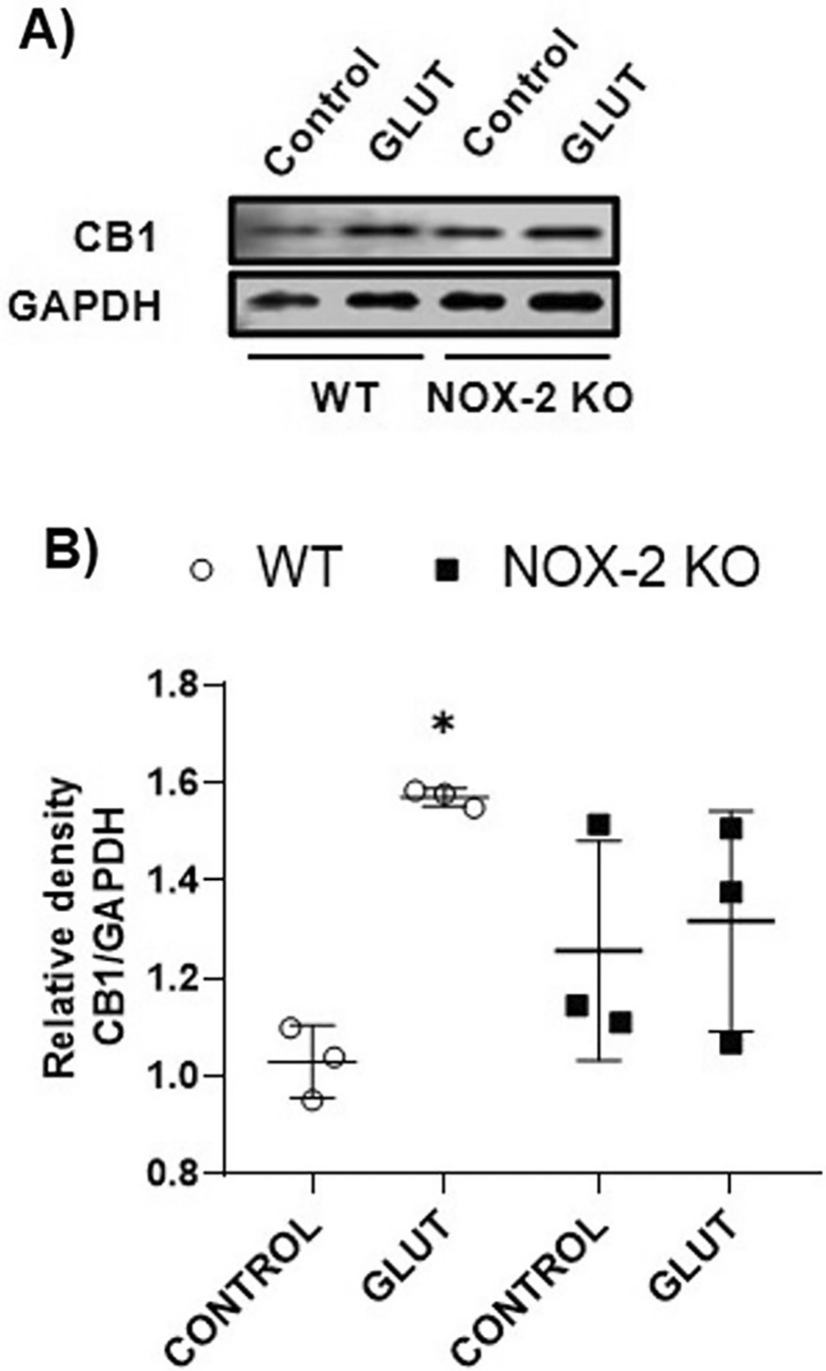
Expression of CB1 in WT and NOX‐2 KO mice after glutamate (GLUT) intracerebral injection at 12 h (A). Western blot of CB1 and GAPDH representative images (*n* = 3). (B). Levels of CB1 at 12 h. Results are expressed as fold over the relative intensity of the load control GAPDH (37 kDa). Values represent the mean ± standard deviation. CB1 (64 kDa). *p < 0.05 versus the control.

In WT animals, we observed that CB1 levels were significantly elevated at 12 h after glutamate intracerebral injection in comparison with the control group. In contrast, in NOX‐2 KO mice, we did not observe any statistically significant difference between the control and glutamate‐treated groups at 12 h (Figure 1A,B).

### 3.2 WIN55 Reduces Motor Dysfunction in WT Mice

**FIGURE 2:**
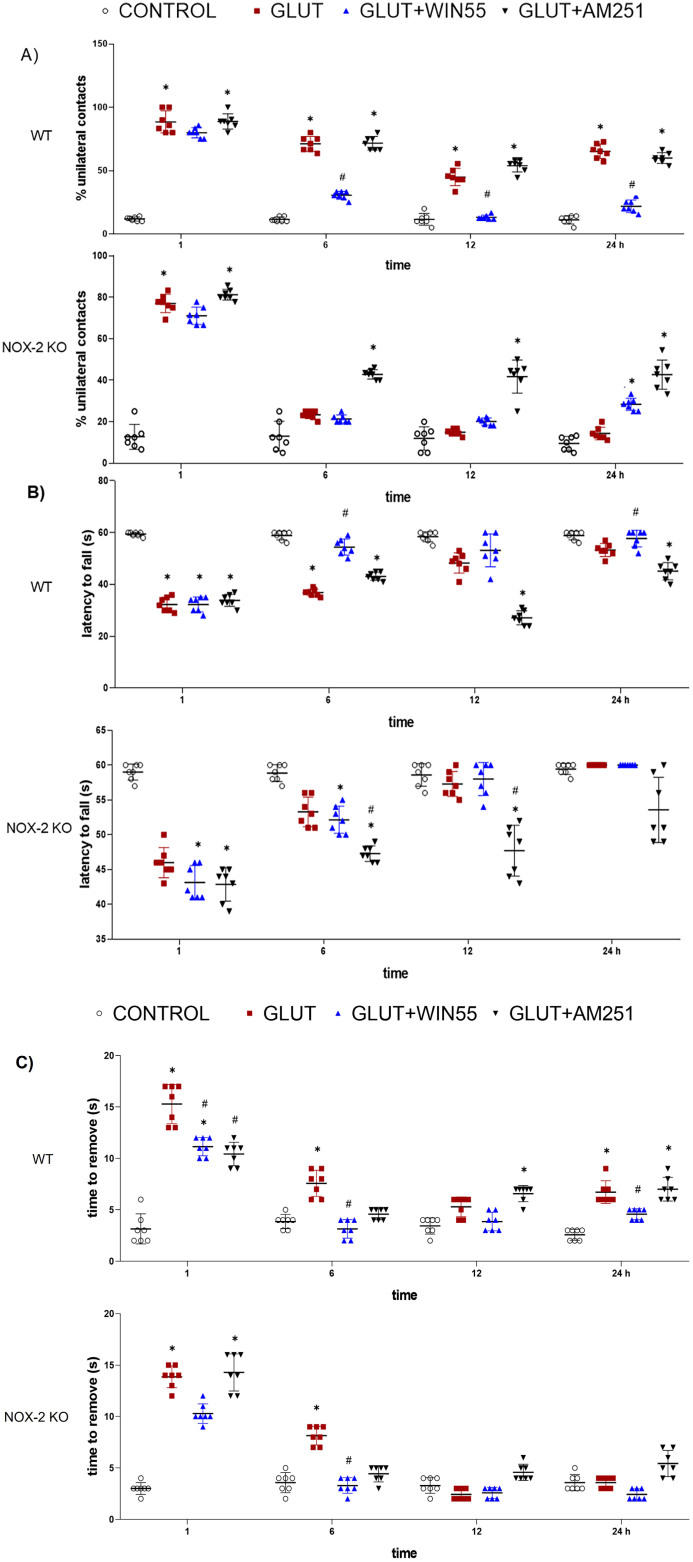
Motor activity recovery after glutamate (GLUT) administration in WT and NOX‐2 KO mice treated with or without WIN55, or AM251 evaluated at 1, 6, 12, and 24 h after intracerebral glutamate (GLUT) injection (*n* = 7). (A) Cylinder test. (B) Latency to fall. (C) Adhesive removal test. Results were expressed as means ± standard deviation. *p < 0.05 versus the corresponding control; #*p* < 0.05 versus the corresponding mice treated with glutamate (GLUT).

In Figure 2A, during the cylinder test, it was observed that WT mice administered glutamate alone, glutamate with AM251, or glutamate in combination with WIN55, all exhibited a comparable rise in the count of one‐sided contacts during the initial hour (88 ± 8% and 88 ± 6%, with a significance of *p* < 0.05). After 12 h, the treatment with glutamate and glutamate plus AM251 decreased the number of unilateral contacts 65 ± 5% and 59 ± 4, respectively (*p* < 0.05); however, animals treated with glutamate plus WIN55 showed a markedly progressive decrease of unilateral contacts from the 6 h as compared to glutamate alone (30 ± 3% vs. 71 ± 5%, respectively, *p* < 0.05) to 24 h (21 ± 4% vs. 65 ± 5%, *p* < 0.05; Figure 2A). In contrast, when NOX‐2 KO animals were treated with glutamate or glutamate plus WIN55, there was a similar decrease in the percentage of unilateral contacts from 6 h (23 ± 1% and 21 ± 1%, respectively, *p* < 0.05) to 24 h (14 ± 3% and 28 ± 2%, respectively, *p* < 0.05). Interestingly, administration of glutamate with AM251 to animals resulted in a notable increase in one‐sided contacts compared to those treated with glutamate alone, showing 42 ± 2% and 47 ± 7% of contacts at 12 and 24 h, versus 23 ± 1% and 14 ± 3%, respectively, as depicted in Figure 2A.

One hour after injury, WT animals treated with glutamate or glutamate plus WIN55 or AM251 were unable to hold to the grid for more than 33 s (32 ± 2 s and 32 ± 2 s and 33 ± 3 s, respectively). The animals treated with glutamate plus WIN55 showed a significative gradual increase in holding time in comparison with animals treated with glutamate at 6 h (54 ± 3 s vs. 36 ± 1 s, respectively, *p* < 0.05) and 24 h (60 ± 0 s vs. 53 ± 2 s, respectively, *p* < 0.05; Figure 2B). The holding time in the glutamate and glutamate plus AM251 groups was shorter at 6 (36 ± 1 s and 43 ± 1 s, respectively), 12 (48 ± 3 s and 27 ± 2 s), and 24 h (53 ± 2 s and 45 ± 3 s). Control mice were held to the grid for 1 m. NOX‐2 KO animals and those administered glutamate or glutamate in combination with WIN55 showed comparable holding times, remaining consistent from 6 h (53 ± 2 s and 53 ± 1 s, respectively) to 24 h (60 ± 0 s for both conditions). In contrast, animals treated with glutamate and AM251 displayed a reduced holding time at 12 h (47 ± 3 s) and 24 h (53 ± 4 s), with both time points showing statistically significant differences (*p* < 0.05).

In WT animals treated with glutamate, adhesive removal times were longer than in those treated with both glutamate and WIN55, with statistically significant differences noted at 1 (15 ± 2 for glutamate alone compared to 11 ± 1 s for combination), 6 h (7 ± 1 s for glutamate alone compared to 3 ± 0 s for the combination, *p* < 0.05), and 24 h (6 ± 1 s compared to 7 ± 1 s, *p* < 0.05). Animals treated with glutamate paired with AM251 exhibited similar removal times to the glutamate‐treated group (Figure 2C). In contrast, in the NOX‐2 knockout group, the combination of glutamate and WIN55 significantly shortened the duration needed for adhesive removal at 6 h (3 ± 0 s), when compared to those treated solely with glutamate at the same time (8 ± 0 s *p* < 0.05) and to those treated with glutamate plus AM251 (4 ± 0 s). At 12 and 24 h, no significant differences were observed in the experimental groups. The control group consistently removed the adhesive in a shorter time.

### 3.3 In WT Animals, WIN55 Attenuated Excitotoxic Injury

**FIGURE 3:**
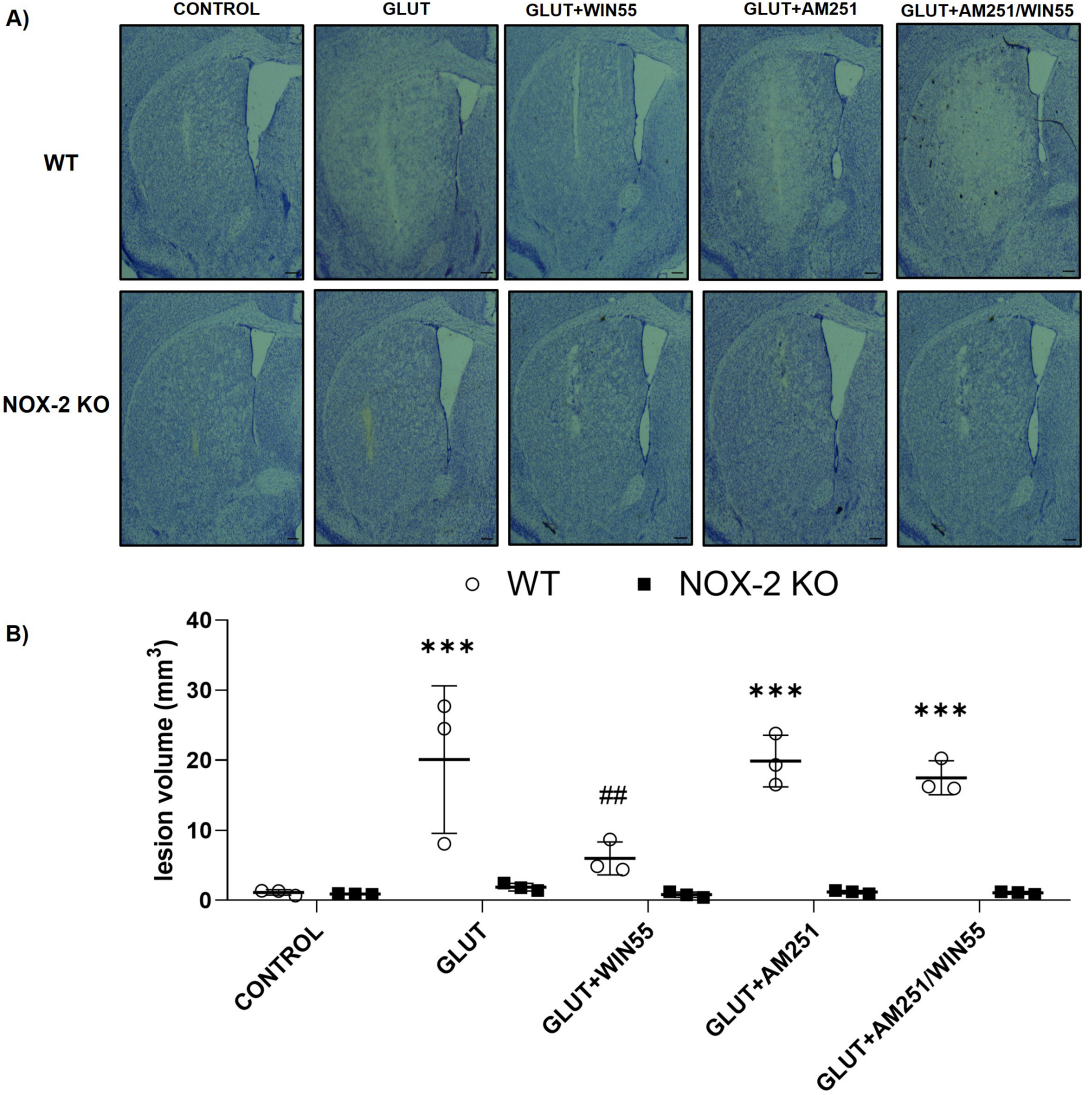
Lesion volume in WT and NOX‐2 KO mice after glutamate intracerebral administration and treated with or without WIN55, AM251, and AM251/WIN55. The lesion was evaluated after 24 h of the intracerebral glutamate (GLUT) injection (*n* = 3). (A) Representative micrographs of coronal striatal sections stained with cresyl violet. The scale bars represent 200 μm. (B) Quantification of the lesion volume is expressed in cubic millimeters. Results were expressed as means ± standard deviation ***p < 0.001 versus the corresponding control; ##*p* < 0.01 versus the corresponding mice treated with glutamate (GLUT).

Twenty‐four hours post‐injury lesion volume was evaluated as shown in Figure 3. WT animals that were injected with glutamate exhibited a substantially larger lesion volume (20.08 ± 10.53 mm^3^, *p* < 0.001) compared to the controls (1.124 ± 0.38 mm^3^). WIN55 treatment significantly reduced the lesion size in glutamate‐treated WT animals (5.98 ± 2.36 mm^3^, *p* < 0.01). However, the lesion volumes in glutamate‐treated WT animals that also received AM251 or a combination of AM251 and WIN55 were comparable to those treated with glutamate alone, measuring 19.88 ± 3.67 mm^3^ and 17.48 ± 2.41 mm^3^, respectively, as depicted in Figure 3. As anticipated, NOX‐2 KO animals with intracerebral glutamate injection showed significantly less lesion volume (1.88 ± 0.53 mm^3^) than WT animals treated similarly. Furthermore, lesions in NOX‐2 KO animals were not notably smaller when animals were also treated with WIN55 (0.802 ± 0.39 mm^3^), AM251 (1.17 ± 0.24 mm^3^), or the combination of AM251 and WIN55 (1.17 ± 0.19 mm^3^); these lesion sizes were similar to control NOX‐2 KO animals (0.908 ± 0.06 mm^3^).

### 3.4 The Brain Edema Was Lower in WIN55‐Treated WT Animals

**FIGURE 4:**
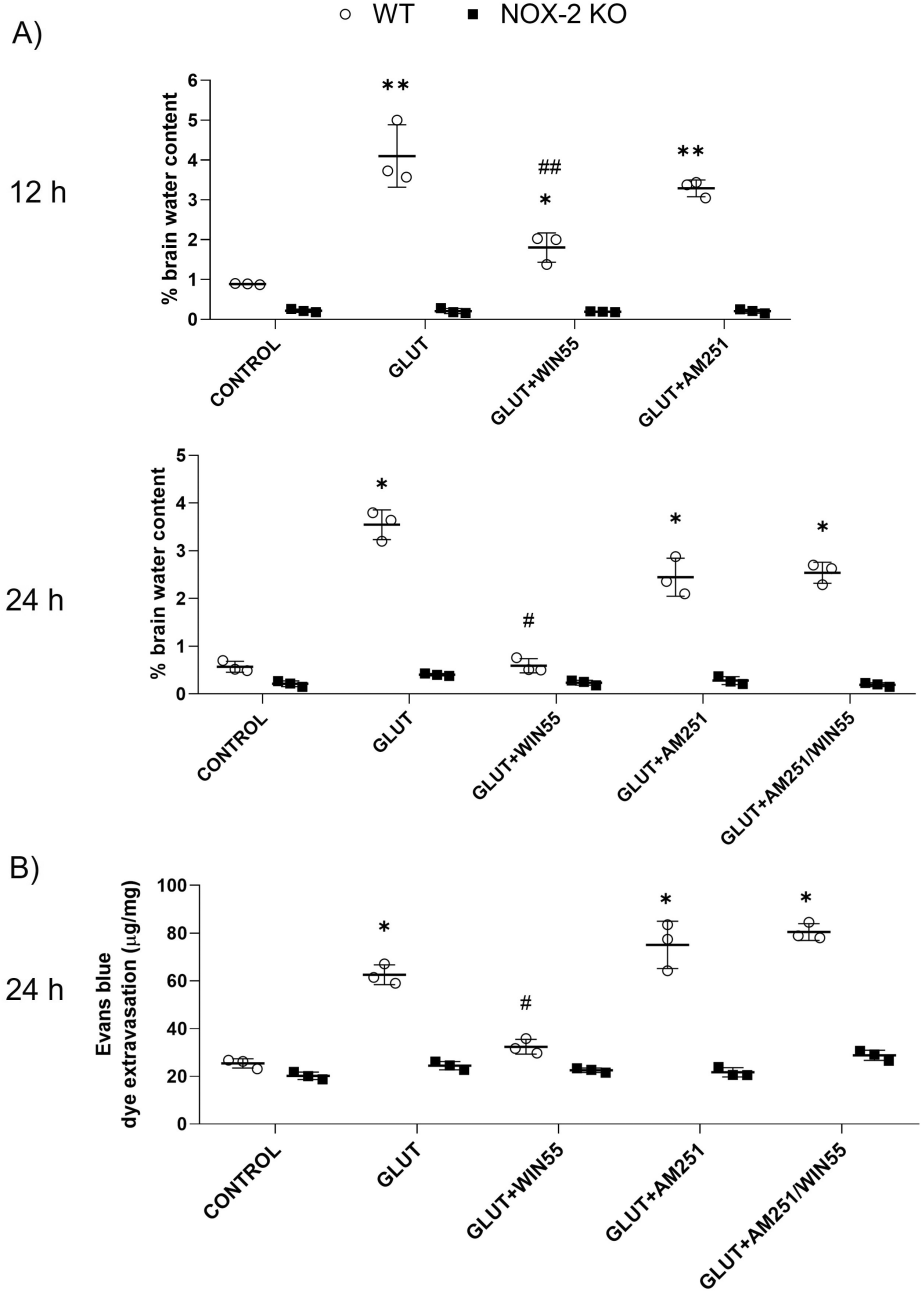
(A) Brain water content in WT and NOX‐2 KO mice at 12 and 24 h after glutamate (GLUT) intracerebral injection and treated with or without WIN55, AM251, or AM251/WIN55 (*n* = 3). (B) Evans Blue leakage quantification at 24 h after glutamate intracerebral injection and treatments (*n* = 3). Results are means ± standard deviation. *p < 0.05, **p < 0.01, versus the corresponding control; #*p* < 0.05, ##*p* < 0.01 versus the corresponding mice treated with glutamate (GLUT).

In WT animals, 12 h after injury, the percentage of brain water after glutamate intracerebral injection increased by 4.1% ± 0.78% (*p* < 0.01) in contrast to the significantly lower water content found in WIN55‐treated animals (1.8% ± 0.36%; *p* < 0.01; Figure 4A). Brain water content in the AM251‐treated group and control animals was 3.29 ± 0.21% and 0.88 ± 0.10%, respectively. In NOX‐2 KO animals, brain water content was not statistically different between the control group (0.21% ± 0.04) and glutamate, glutamate plus WIIN55, or glutamate plus AM251 animals (0.20 ± 0.06, 0.19 ± 0.01 and 0.20 ± 0.05%, respectively). After 24 h, the mean brain water content in WT control animals was 0.57 ± 0.11%, while the water content in glutamate‐treated mice increased to 3.54 ± 0.31%, which was significantly decreased in the WIN55 group (0.59 ± 0.14; *p* < 0.05). AM251 treatment did not significantly reduce the water content induced by glutamate (2.44 ± 0.39%), but markedly reduced the observed effect of WIN55 (2.54 ± 0.21%; *p* < 0.05; Figure 4A). In NOX‐2 KO mice, the brain water content in the control was 0.21 ± 0.06, which increased to 0.40 ± 0.02% in glutamate‐treated animals. In contrast to the WIN55 treatment (0.23 ± 0.05%), the AM251 treatment (0.28 ± 0.08%) did not modify the observed increase of water content induced by glutamate (Figure 4A).

### 3.5 WIN‐55 Prevented the BBB Disruption in WT Mice

After 24 h, in WT groups, Evans blue leakage in glutamate‐treated mice significantly increased (62.53 ± 4.14 μg/mg, *p* < 0.05) in contrast to the control group (25.39 ± 1.95 μg/mg). The observed action of glutamate was significantly reduced by WIN55 (32.39 ± 3.10 μg/mg, *p* < 0.05), which was no longer observed in the presence of AM251 (80.47 ± 3.52 μg/mg). The effect of glutamate treatment was not modified by AM251 alone (75.10 ± 9.88 μg/mg; Figure 4B). In NOX‐2 KO animals, Evans blue leakage was not statistically different between the control group (20.21 ± 1.54 μg/mg) and glutamate, WIN55 or AM251‐treated groups (24.48 ± 1.73 μg/mg, 22.55 ± 0.94 μg/mg, and 21.70 ± 1.93 μg/mg, respectively). Dye leakage increased significantly in the AM251/WIN55‐treated mice (28.78 ± 2.12 μg/mg, *p* < 0.05).

### 3.6 AQP4 Level Was Diminished in WIN55‐Treated Animals

**FIGURE 5:**
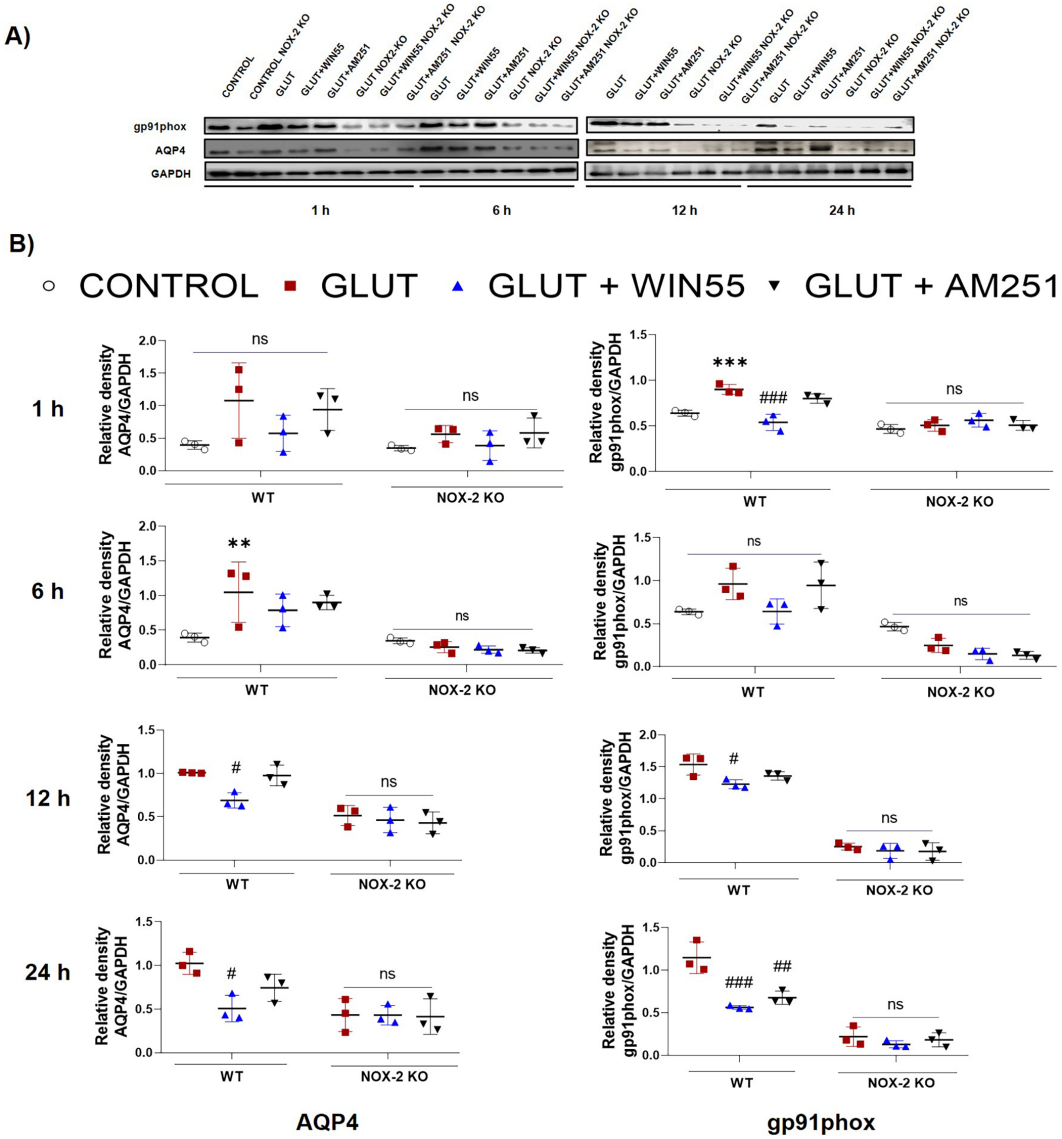
Striatal protein levels of gp91phox and AQP4 in WT (WT) and NOX‐2 KO mice after 1, 6, 12 and 24 h of glutamate (GLUT) intracerebral injection with or without WIN55 or AM251. (A) Western blot of gp91phox, AQP4, and GAPDH representative images (*n* = 3). (B) Levels of gp91phox and AQP4 at 1,6,12, and 24 h. Results are expressed as fold over the relative intensity of load control GAPDH (37 kDa). Values represent the average ± standard deviation. gp91phox (60/91 kDa), AQP4 (39 kDa). *p < 0.05, **p < 0.01, ***p < 0.001 versus the corresponding control; #*p* < 0.05, ##*p* < 0.01, ###*p* < 0.001 versus the corresponding mice treated with glutamate (GLUT).

In WT animals, levels of the AQP4 protein increased from 6 to 24 h after administration of glutamate. A notable decrease in AQP4 protein levels was observed at 12 and 24 h in animals treated with WIN55. However, this reduction was not evident when the animals were treated with AM251. Administration of AM251 by itself had no impact on the increase in AQP4 observed in mice treated with glutamate (as shown in Figure 5A,B). In NOX‐2 KO animals, AQP4 expression levels did not differ significantly between any of the groups throughout the 1–24 h timeframe (illustrated in Figure 5A,B).

### 3.7 NOX‐2 Catalytic Subunit gp91^phox^ Level Was Reduced by WIN55

At 1, 12, and 24 h, the gp91^phox^ protein levels were elevated in glutamate‐treated WT mice compared to protein levels observed in the control group. This effect was significantly reduced in animals treated with WIN55 at all times evaluated. AM251 partially reduced the impact of glutamate (Figure 5A,B). In NOX‐2 KO mice, protein levels were not statically different in all groups (Figure 5A,B).

### 3.8 WIN‐55 Reduced NOX Activity in WT Animals

**FIGURE 6:**
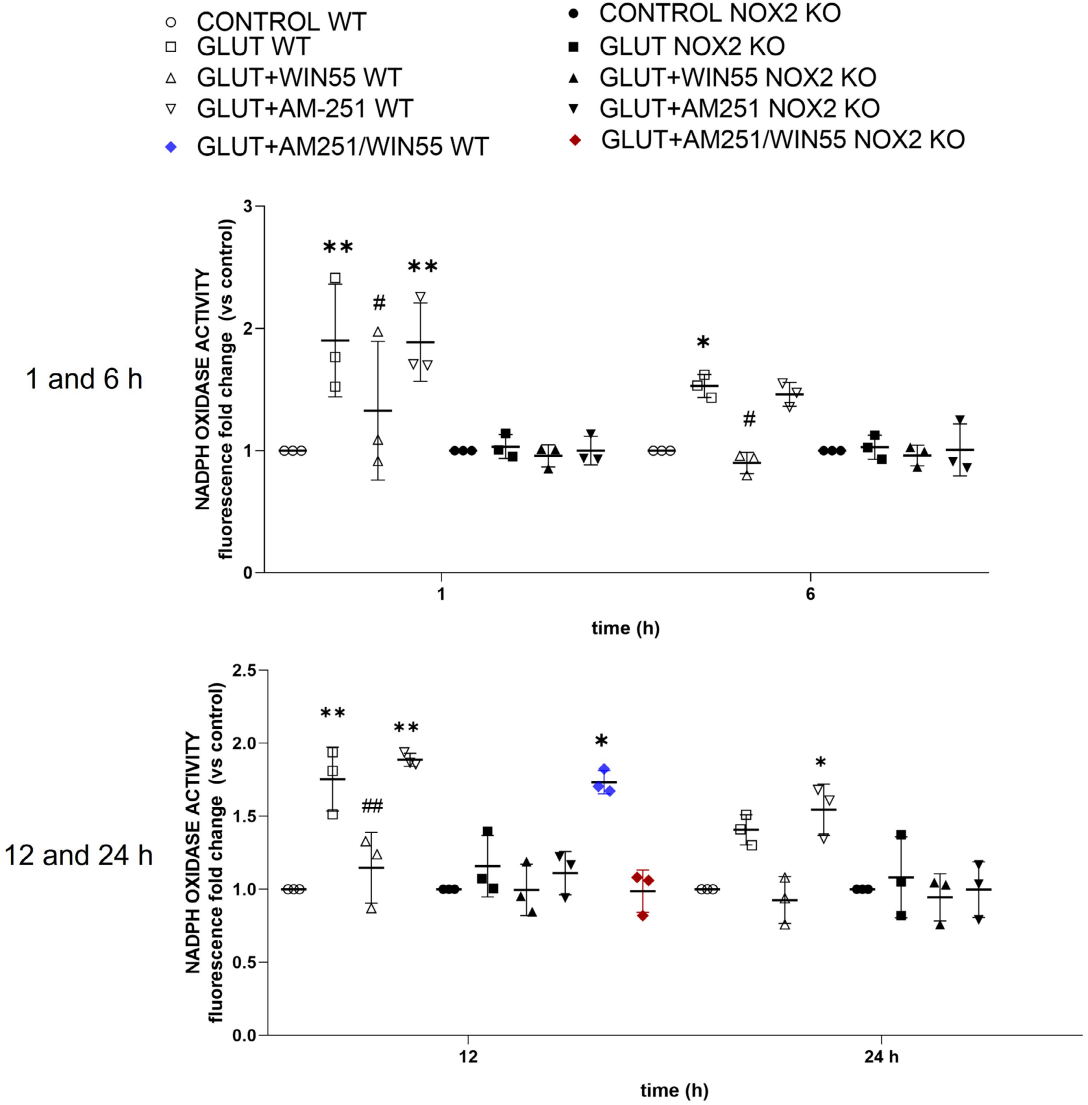
Striatal NOX activity during excitotoxicity in wild‐type (WT) and NOX‐2 KO mice after glutamate (GLUT) intracerebral injection treated with or without WIN55, AM251, and AM251/WIN55 at 1,6,12, and 24 h (*n* = 3). Data are expressed as fold change of Et fluorescence relative to controls. Values were expressed as means ± standard deviation. *p < 0.05, **p < 0.01 versus the corresponding control; #*p* < 0.05, ##*p* < 0.01 versus the corresponding mice treated with glutamate (GLUT).

In WT animals, in comparison with the control group, striatal NOX activity was significantly elevated at 1 (1.90 ± 0.46, *p* < 0.01), 6 (1.52 ± 0.09, *p* < 0.05), and 12 h (1.75 ± 0.21, *p* < 0.001) in glutamate‐treated mice. These values were not significantly different when animals were additionally treated with AM251 (1.88 ± 0.32, 1.46 ± 0.09, and 1.88 ± 0.04‐fold, respectively). In contrast, the enzyme activity levels were decreased with WIN55 treatment at 1 (1.32 ± 0.56, *p* < 0.05), 12 (1.14 ± 0.24, *p* < 0.05), and 24 h (0.92 ± 0.16‐fold, respectively, *p* < 0.01). The effect of WIN55 treatment was noticeably diminished when animals were concurrently treated with AM‐251, in the group receiving both AM251 and WIN55. NOX activity demonstrated a significant elevation (1.73 ± 0.08, *p* < 0.05, at 12 h) as presented in Figure 6. In contrast, NOX activity levels in NOX‐2 KO mice remained statistically indistinguishable among the control group and all treatment groups across the entire 1–24‐h period, as illustrated in Figure 6.

### 3.9 WIN55 Attenuated the ROS Production During Excitotoxicity

**FIGURE 7:**
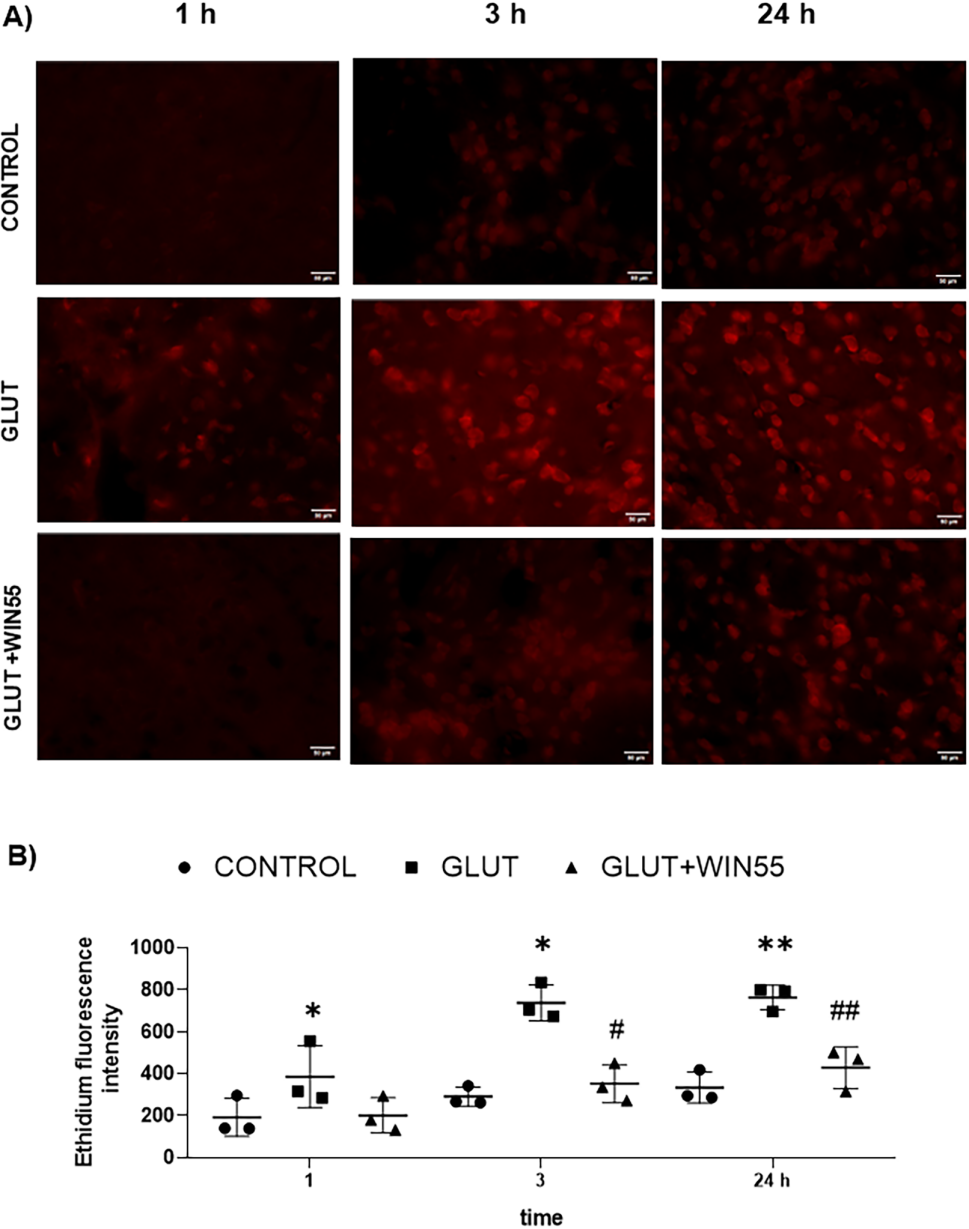
Reactive oxygen species (ROS) production in WT mice after glutamate (GLUT) intracerebral injection treated with or without WIN55, at 1, 3, and 24 h. (A) Representative microphotographs of DHE staining of the striatum (*n* = 3). The scale bar represents 50 μm. (B) Quantification of Ethidium fluorescence intensity in the different groups. Results are means ± standard deviation. *p < 0.05, **p < 0.01, versus the corresponding control; #*p* < 0.05, ##*p* < 0.01 versus the corresponding mice treated with glutamate (GLUT).

The formation of ROS in the striatum was quantified through the oxidation of DHE to Et at 1, 3, and 24 h after glutamate injection in WT animals (Figure 7A). ROS levels were significantly elevated after glutamate intracerebral injection in comparison with control mice at 1 h (385.23 ± 147.86 vs. 191.61 ± 90.75, respectively, *p* < 0.05), 3 h (737.89 ± 85.40 vs. 290.14 ± 44.96, respectively, *p* < 0.05), and 24 h (763.44 ± 58.29, respectively, *p* < 0.01). The treatment with WIN55 significantly reduced ROS production induced by glutamate at 3 h (352.48 ± 90.05, *p* < 0.05) and 24 h (428.29 ± 99.13, *p* < 0.01) (Figure 7B).

### 3.10 NF‐κB p65 Protein Level Was Reduced in WIN55‐Treated WT Animals

**FIGURE 8:**
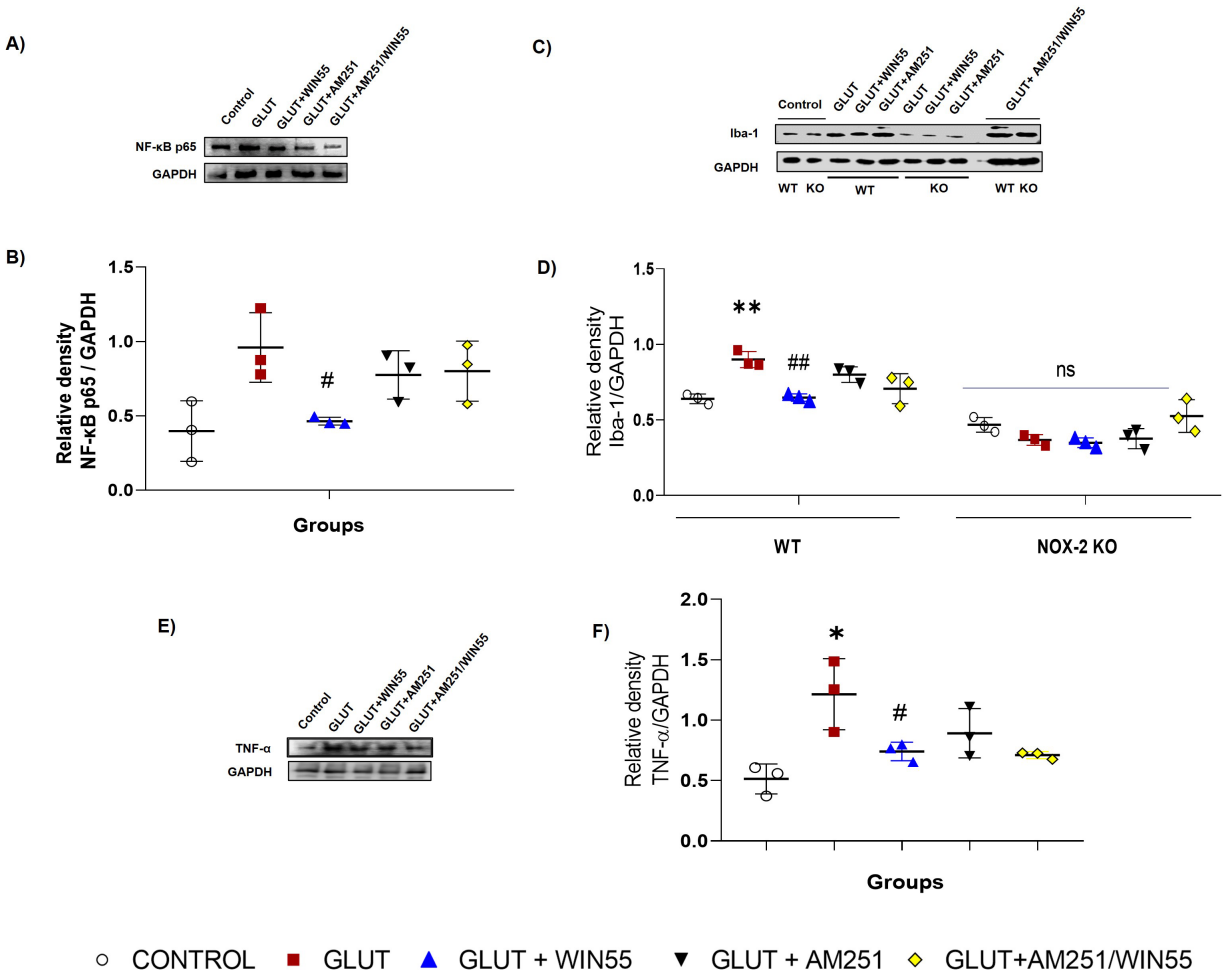
Protein levels of Iba‐1, TNF‐α, and NF‐κB p65 in mice at 12 h after glutamate (GLUT) intracerebral injection and treated with or without WIN55, AM251, and AM251/WIN55. (A) Representative Western blot of Iba‐1 in WT and NOX‐2 KO mice. (B) Levels of Iba‐1 (*n* = 3). (C) Representative Western blot of TNF‐α in WT mice. (D) Levels of TNF‐α (*n* = 3). (E) Representative Western blot of NF‐κB p65 in WT mice. (F) Levels of NF‐κB p65 at (*n* = 3)Values represent the average ± standard deviation. Iba‐1 (15 kDa), TNF‐α (26 kDa), NF‐κB p65 (65 kDa). *p < 0.05, **p < 0.05, ##*p* < 0.01 versus the corresponding saline control; #*p* < 0.05 versus the corresponding mice treated with glutamate (GLUT).

Protein levels of NF‐κB were assessed at 12 h to evaluate the participation of this transcription factor in the neuroprotective effects of CB1 stimulation. Figure 8A,B shows that glutamate intrastriatal injection markedly increased the levels of NF‐κB p65. These levels were significantly reduced by WIN55 treatment.

### 3.11 WIN55 Attenuated Iba‐1 and TNF‐α Levels During the Excitotoxic Injury

To evaluate the involvement of neuroinflammation in the CB1 neuroprotective actions, the protein levels of Iba‐1 and TNF‐α were analyzed at 12 h after injury. Figure 8C,D shows that WIN55 administration attenuated increases in Iba‐1 triggered by glutamate intracerebral injection in WT mice. In NOX‐2 KO animals, Iba‐1 protein levels were not statically different between the control group and the rest of the groups. In the same way, after excitotoxicity, TNF‐α level increased in comparison with control, though, the administration of WIN55 reduced the protein level of this pro‐inflammatory cytokine in WT animals (Figure 8E,F).

## 4 Discussion

Excitotoxicity is a severe phenomenon usually secondary to stroke, TBI, and neurodegenerative diseases that cause damage to the nervous system cells [39, 40]. The excessive activation of NMDA and AMPA receptors is responsible for an increase in intracellular Ca^2+^, and thus the activation of intracellular pathways related to neuronal damage [41]. NMDA receptor stimulation activates NOX and produces ROS [42] suggesting that Ca^2+^ influx through the NMDAR channel is crucial for NOX2 activation, while alternative pathways do not lead to NOX2 activation or neuronal death. This signaling links NMDAR activation to downstream effects, starting with Ca^2+^ influx that activates PI3K and PKC [43]. Glutamate is the most important excitant amino acid implicated in excitotoxicity and increases in extracellular levels have been reported at 1, 6, 24, and 48 h after TBI, suggesting the participation during brain injury [44, 45, 46].

The ECS is linked to brain damage, with the CB1 receptor being crucial. We found that CB1 expression in the striatum increased after glutamate injection in WT animals, likely enhancing endogenous cannabinoid effects in pathological conditions. Increased CB1 receptor mRNA levels were noted in a kainic acid‐induced excitotoxicity model 24 h postinjection [47]. Zoppi et al. [48] reported that in animals exposed to chronic stress, the NMDA‐selective blocker MX‐801 significantly reduced CB1 protein levels, suggesting that CB1 upregulation during excitotoxicity is a compensatory response to glutamate release, aimed at reducing neuronal damage. Similar increases in CB1 receptors were observed in the hippocampus after chronic intermittent hypoxia (CIH) [49] and in an ischemic stroke model [50]. In NOX‐2 KO animals, the CB1 protein levels were not modified after glutamate intracerebral injection, it is probably that reduction in brain damage due to NOX‐2 deficiency limited the response of ECS, at least at the CB1 level, and suggests the participation of this receptor in injury conditions.

In the present study, we observed that the administration of glutamate via intrastriatal injection caused significant damage to the striatal region and impaired motor function. Interestingly, when we stimulated the CB receptors by the administration of WIN55, we observed a marked improvement in muscular strength and voluntary movement, in contrast to the CB1 antagonist AM251 alone, which also completely abolished the WIN55 effects. Consistent with these findings, it has been reported that the increase in endogenous anandamide levels by using a fatty acid amide hydrolase inhibitor, PF04457845, improves motor function in a TBI mice model [51].

We have previously demonstrated that mice with excitotoxic damage in the striatum exhibited compromised sensorimotor abilities [7]. In the current investigation, it was observed that, in WT mice, treatment with WIN55 decreased the size of the lesion and further verified its association with motor improvement. These observations, especially in mice treated with CB1 antagonists, underscore the role of the CB1 receptor in excitotoxic processes. Additionally, the contribution of NOX‐2 to both the reduction in lesion size and the accelerated motor recovery post‐glutamate injection was validated in NOX‐2 KO mice.

In contrast, to what was observed in WT mice, the absence of NOX‐2 induced significantly lower motor deficits after striatal glutamate injection. In most of the motor tests, in NOX‐2 KO mice, the treatment with WIN55 in glutamate‐injected animals showed a similar effect as in glutamate‐treated mice, suggesting the participation of NOX‐2 in the neuroprotective activity by endocannabinoid receptor agonist. In these animals, the treatment with the antagonist AM251 induced a significant delay in motor alteration recovery, which could be due to an interaction with another intracellular NOX‐2‐independent signaling pathway associated with death and /or neuronal survival [52, 53].

The increase in NOX‐2 activity is critical in the glutamate‐induced excitotoxicity damage and is related to the toxic intraneuronal accumulation of calcium that leads to excessive ROS production [54]. Elevated NOX activity has been reported at 6 and 24 h following ischemic/reperfusion brain injury [55, 56]. Additionally, Ansari et al. reported an increase in NOX activity from 6 to 72 h in rats subjected to cortical contusion. Our results evidenced an increase in the NOX‐2 catalytic subunit gp91phox protein levels and the NOX activity after glutamate intracerebral injection that was reversed by WIN55 administration. It is important to acknowledge that NOX activity assay is not specific to NOX‐2; however, it is a good parameter to estimate their activity. This effect was completely abolished when CB1 was blocked before or after brain injury, thus reinforcing the relevance of CB1 in excitotoxicity. In this regard, Chung et al. [57] reported that WIN55 reduces the expression of p47phox and Rac‐1, two cytosolic components of NOX‐2, in an MPTP‐induced neurotoxic animal model; however, changes in catalytic subunit gp91phox expression and NOX activity in the first 24 h after excitotoxic damage has not been reported.

Increases in oxidative stress during excitotoxicity were previously reported at 12 h after excitotoxic damage [6]. In the present study, we reported an increase in ROS formation from 1 h up to 24 h after excitotoxic damage. The stimulation of the endocannabinoid receptor reduced ROS formation from 3 h up to 24 h and this result demonstrated a correlation with the reduction in NOX activity and NOX‐2 catalytic subunit expression. Although other ROS sources during brain injury should be considered, NOX‐2 plays a critical role in excitotoxicity [6].

It has been reported that AQP4 increase is induced by oxidative stress and engages in edema formation after brain injury [14, 58]. While the cannabinoid receptors and AQP4 expression are not directly related following TBI [59], recent findings indicate that post‐traumatic declines in plasma concentrations of the endocannabinoids 2‐AG and AEA are associated with an increase in cerebral edema and AQP4 expression. This suggests that the ECS is crucial in regulating AQP4 expression and maintaining water homeostasis in the brain [60]. The findings of our study indicate that activation of the cannabinoid receptor leads to a decrease in AQP4 protein levels following intracerebral injection of glutamate, corroborating the outcomes documented by Jiang and colleagues [61]. Oxidative stress participates in AQP4 expression and cellular localization in astrocytes [14]. The increase in ROS in neuron culture after NMDA treatment is observed at 25 min [42]. Therefore, it is possible that ROS produced by NOX‐2 may be responsible for the increase in total AQP4 levels observed at those times, as demonstrated by Bi et al. [14]. Reduction in ROS formation after WIN55 treatment coincides with a reduction in AQP4 expression, reducing BBB disruption and edema formation. We did not identify an increase in AQP4 protein levels in NOX‐2 KO mice after glutamate injection, corroborating that ROS produced by NOX‐2 participates in AQP4 expression after excitotoxic damage.

Excitotoxicity increases water retention in the brain, which accelerates the development of vasogenic edema. As mentioned above, oxidative stress stimulates AQP4 translocation and facilitates water influx concerning changes in the ionic balance. Excitotoxic damage increases glutamate reuptake through excitatory amino acid transporters EEAT1 and EEAT2 favoring neuron and astrocyte swelling. The increase in Ca^2+^ influx exacerbates water influx through AQP4 and causes early BBB disruption and neuronal death [9]. In the present study, we observed that WIN55 significantly reduced brain edema after glutamate injection. The early treatment with the endocannabinoid receptors agonist may inhibit glutamate release and, therefore, a decrease of Ca^2+^ influx and water entry. The above‐mentioned effect has been reported in ischemic and TBI [20, 61]. The reduction in AQP4 levels by WIN55 limited the water influx and prevented BBB disruption.

In previous reports, the inhibition of 2‐AG hydrolysis reduces BBB permeability after focal ischemic injury in mice [62], an effect previously reported with the treatment of 2‐AG and CBD after TBI [20, 61]. Our results demonstrated that treatment with WIN55 prevents BBB disruption caused by glutamate‐induced excitotoxicity and reduced dye extravasation. In NOX‐2 KO mice, brain water content and Evans blue dye extravasation after excitotoxicity were low in comparison with WT mice and were not modified by any treatment, confirming that oxidative stress participates in the physiopathology of edema and BBB disruption; the decrease of ROS production in NOX‐2 KO mice prevented BBB damage.

NF‐κB is a transcription factor associated with oxidative stress and neuroinflammation after brain injury, and its activation increases NOX‐2 expression in vitro and in vivo models [63, 64]. Some studies have indicated elevated levels of NF‐κB and NOX‐2 proteins in mice after 7 days of poststroke [65]. After excitotoxic damage, an increase in NF‐κB p65 levels was observed, which was reversed by WIN55. This suggests the involvement of NF‐κB in the reduction of NOX‐2 protein levels, NOX activity, and ROS production. The reduction in NF‐κB protein levels after WIN55 treatment has been previously reported in rats subjected to chronic cerebral hypoperfusion (CCH) [66]. Similar results were reported in mice subjected to TBI and treated with the endocannabinoid 2‐AG [21], and in microglial cells exposed to lipopolysaccharide and treated with phytocannabinoids [67]. These results underscore the involvement of NF‐κB in the antioxidant and anti‐inflammatory responses facilitated by WIN55.

Neuroinflammation is a consequence of excitotoxicity and is a significant component of neurodegenerative and acquired brain diseases [68]. In this context, the activation of microglia is critical to the neuroinflammatory process, primarily due to the release of neurotoxic factors such as interleukin‐1 beta (IL‐1β) and tumor necrosis factor‐alpha (TNF‐α), which contribute to progressive neuronal damage [69]. Iba‐1 is recognized as a marker for active microglia and is known to increase in response to brain injury [70, 71]. A reduction in TNF‐α levels following WIN55 treatment has been previously reported in rats with CCH [66]. Similarly, treatment with 2‐AG in mice subjected to TBI decreases levels of this cytokine [21]. These findings suggest that WIN55 mitigates glutamate‐induced neuroinflammation through the reduction of NF‐κB levels.

The regulation of NOX‐2 protein level and NOX activity after WIN55 treatment did not demonstrate significant changes in NOX‐2 KO mice and confirmed the participation of this enzyme in endocannabinoid receptor‐mediated neuroprotection. The role of the transcription factor NF‐κB in the downregulation of NOX‐2 and attenuation of oxidative stress was investigated in WT animals. This led to a decrease in oxidative stress, which in turn inhibited the expression of AQP4 and prevented BBB disruption, edema formation, and neuronal damage. Although WIN55 is a nonspecific agonist for CB1 and CB2, the involvement of the CB1 receptor was corroborated with AM251, which reversed the effects obtained with the agonist activation and confirmed their neuroprotective role in the excitotoxicity [24, 72].

The intracerebral injection of glutamate induces a dysregulated stimulation of the NMDA receptor, which results in an elevated intracellular calcium level in neurons [73]. This condition induces an alteration of mitochondrial homeostasis, as well as a deregulated activation of NOX‐2 that leads to a condition of oxidative stress. On the other hand, it has been reported that NMDA receptor overstimulation activates NF‐κB in cultured cerebellar granule neurons [74], probably mediated by a calcium increase induced by NMDA receptor activation [75] and/or ROS [76]. NF‐κB is a critical factor for the immune response and inflammation. All these events lead to neuronal death.

**FIGURE 9:**
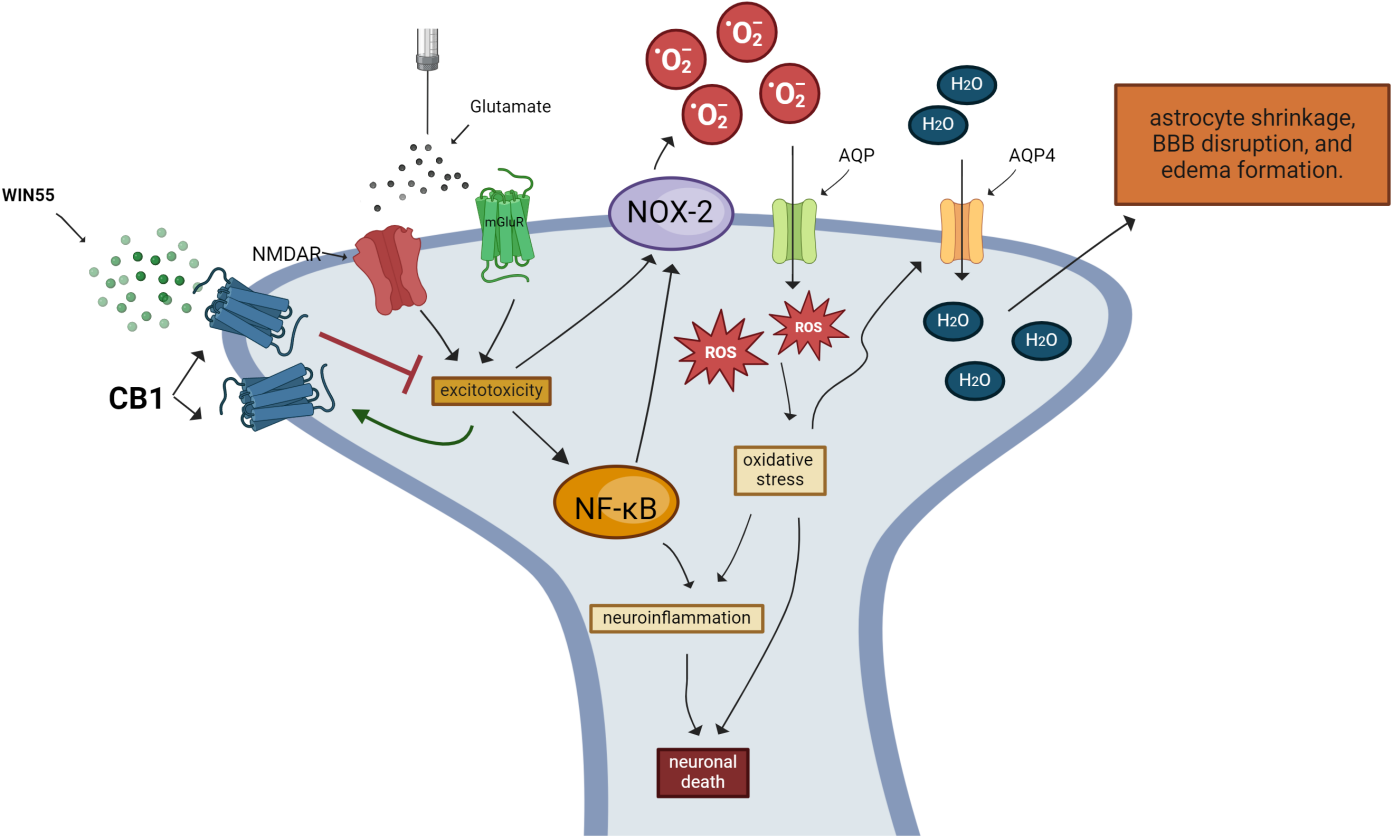
Molecular mechanism implicated in the neuroprotective action of WIN55 in the excitotoxicity induced by glutamate. WIN55 reduces neuronal death, neuroinflammation, oxidative stress, and NOX‐2 activity. The action of the CB1 receptor must be located upstream of all these processes. Glutamate induces the expression of the CB1R that could reinforce the neuroprotective action of WIN55. In astrocytes, the decline in ROS production reduces AQP4 levels and attenuates BBB disruption as well as edema formation.

Our results showed that the activation of the CB1 receptor with WIN55, markedly reduced cell death, inflammatory parameters, ROS levels, and the activation of NOX‐2; therefore, the action of the CB1 receptor must be located upstream of all these processes. We consider that this action occurs very early because ROS production by NOX‐2 activation is already present after 25 min of NMDA receptor activation [42]. We, therefore, propose that some possible early events that CB1 could regulate include increased calcium and loss of mitochondrial homeostasis, as well as NOX‐2 activation (Figure 9).

Overall, our results suggest that the activation of the CB1 receptor by WIN55 exerts a neuroprotective effect in the excitotoxicity induced by glutamate through a reduction in ROS production by a regulation of NOX‐2 activity. Further analysis is necessary to confirm the therapeutic advantage of the endocannabinoid receptors in excitotoxicity.

## Author Contributions

A.M.M.‐T. conducted the experiments, participated in the design of the study, and contributed to the analysis and interpretation of the data, as well as to the writing of the manuscript. J.M. participated in the design of the study, coordinated the study, raised funds, and contributed to the writing of the manuscript. All authors have read and approved the submitted version of the manuscript.

## Ethics Statement

This study was carried out following the accepted standards of animal care and with the procedures approved by the local Animal Care and Use Committee of the Instituto de Fisiología Celular, Universidad Nacional Autónoma de México (protocol number JMA 182‐22). The protocol used followed the Guidelines for the Care and Use of Mammals in Neuroscience as well as guidelines released by the Mexican Institutes of Health Research and the National Institutes of Health Guide for the Care and Use of Laboratory Animals (NIH Publication No. 8023, revised 1978). All efforts were made to minimize animal suffering and to reduce the number of animals used.

## Conflicts of Interest

The authors declare that the research was conducted without any commercial or financial relationships that could be construed as a potential conflict of interest.

## Supporting information


Data S1.

## Data Availability

The raw data supporting the conclusions of this manuscript will be made available by the authors, without undue reservation, to any qualified researcher.
