# Supplementary material for: CB1 Receptor Activation Provides Neuroprotection in an Animal Model of Glutamate‐Induced Excitotoxicity Through a Reduction of NOX‐2 Activity and Oxidative Stress
Source: CNS Neurosci Ther. 2024 Nov 4;30(11):e70099. doi: 10.1111/cns.70099 (PMC11534500; doi:10.1111/cns.70099)
Supplement: Supplementary file 1 — Data S1. [file CNS-30-e70099-s001.pdf]

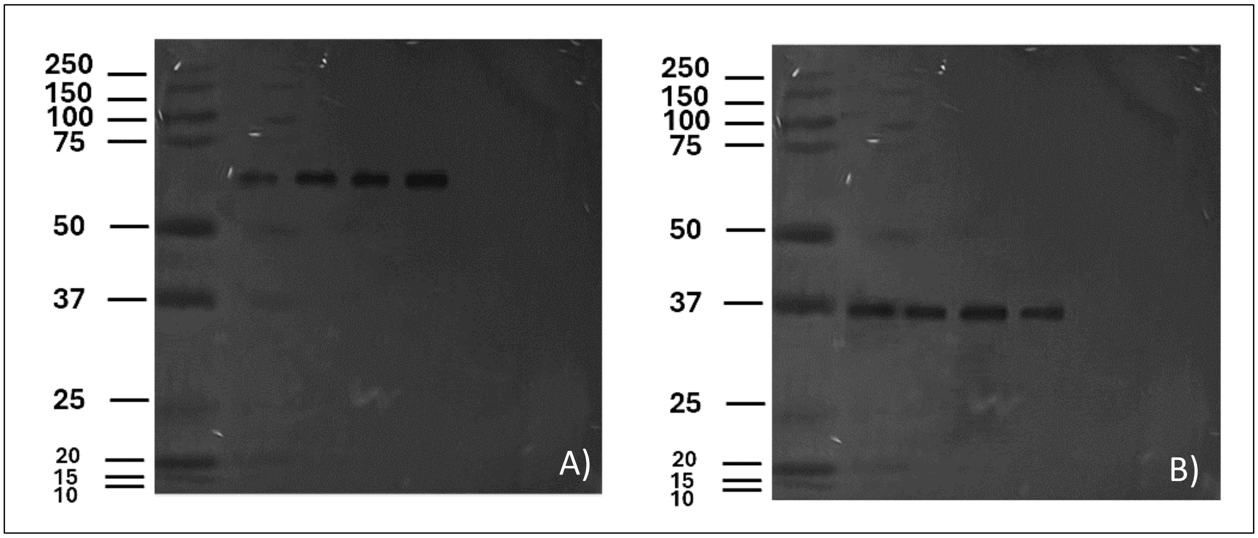

Full unedited blot for Figure 1. CB1 (A) and GAPDH (B).

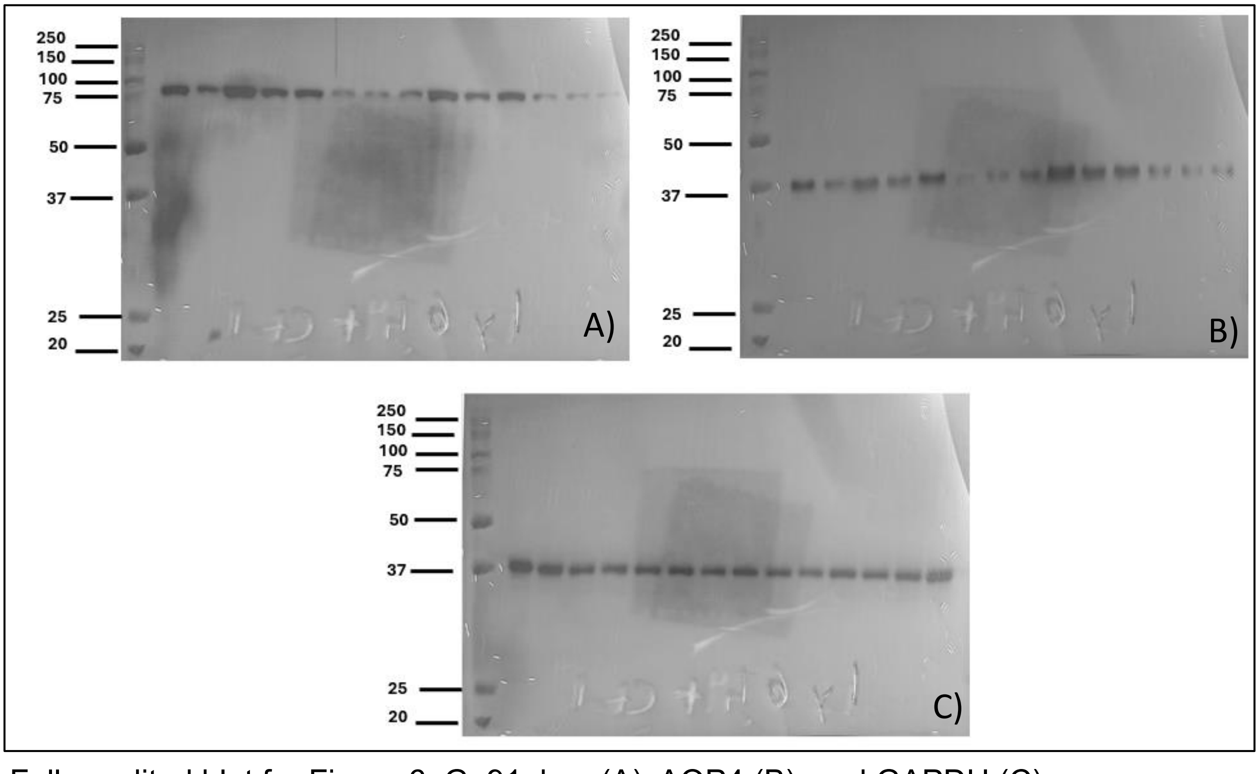

Full unedited blot for Figure 6. Gp91phox (A), AQP4 (B), and GAPDH (C).

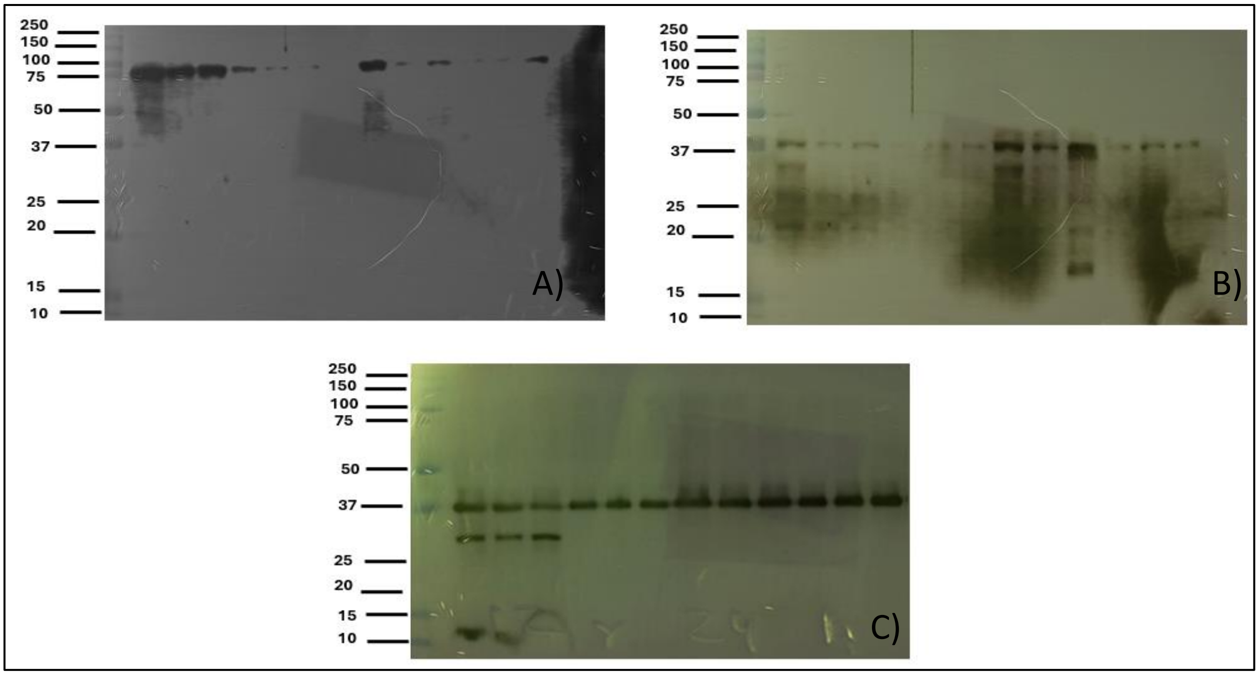

Full unedited blot for Figure 6, Gp91phox (A), AQP4 (B), and GAPDH (C).

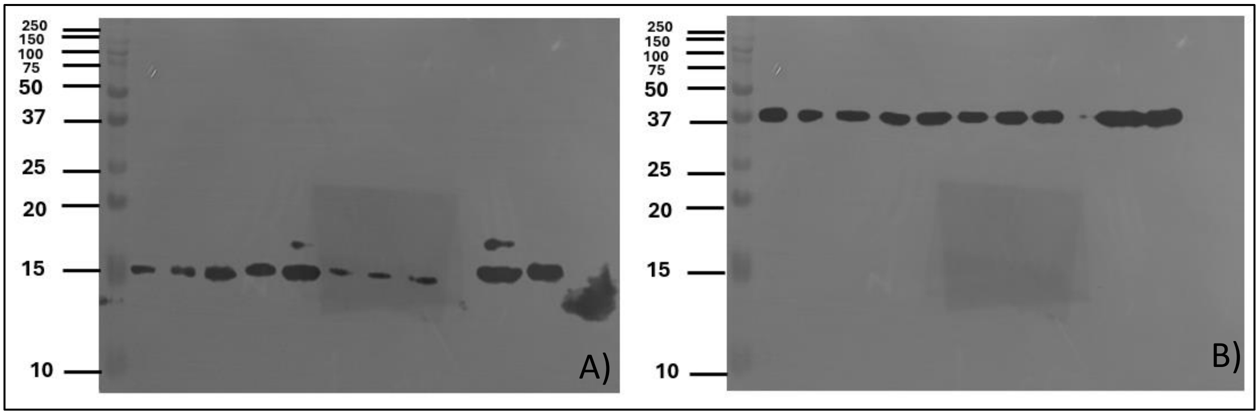

Full unedited blot for Figure 9. Iba-1 (A) and GAPDH (B).

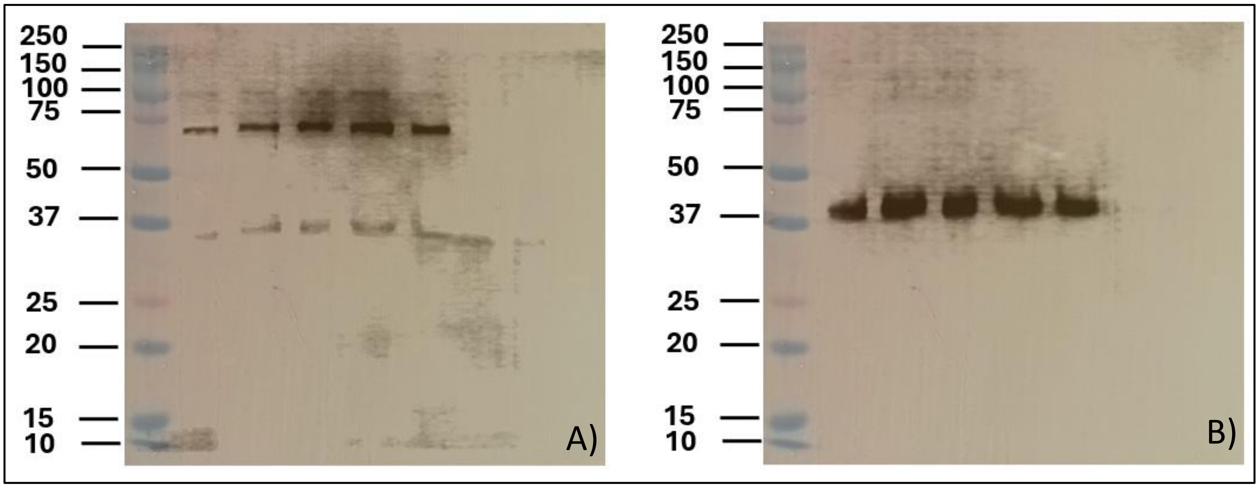

Full unedited blot for Figure 9. NF-κB (A) and GAPDH (B).

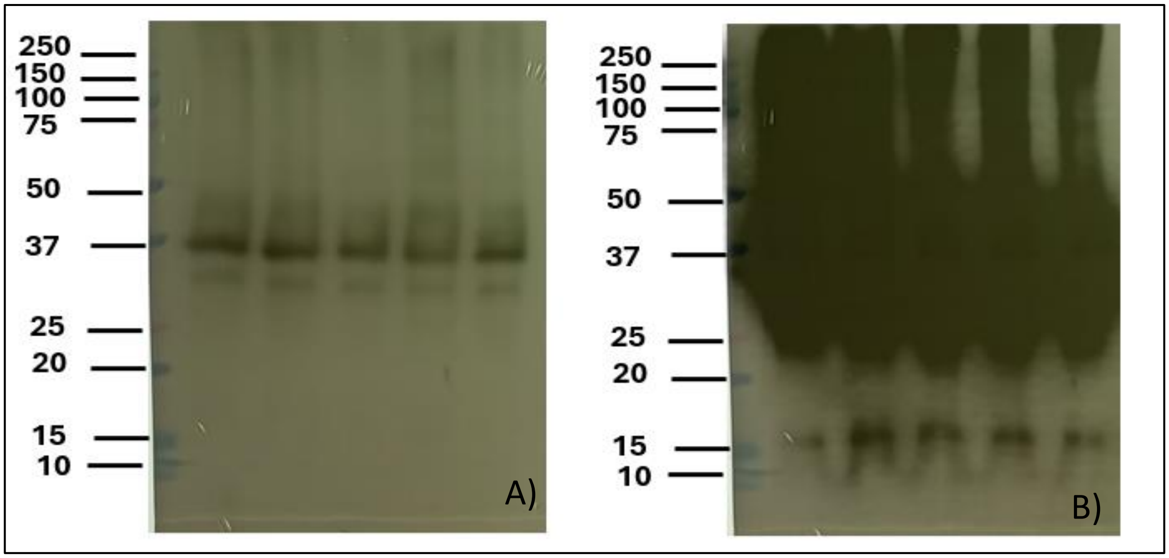

Full unedited blot for Figure 9. TNF-α (A) and GAPDH (B).
